# Audit of Seclusion: Evaluating Performance Against National and Local Standards

**DOI:** 10.1192/bjo.2025.10611

**Published:** 2025-06-20

**Authors:** Sadia Tabassum Javaid, Raman Mittal, Qammar Jabbar, Corrina Bentley, Samantha Dawson

**Affiliations:** North Staffordshire Combined Healthcare NHS Trust, Stoke-on-Trent, United Kingdom

## Abstract

**Aims:** Seclusion, under the Mental Health Act (MHA 1983) Code of Practice, is the supervised confinement of a patient to manage severe risks. The Mental Health Law Governance Group commissioned a clinical audit to assess compliance with the Trust’s Seclusion Policy and MHA Code of Practice, prioritised in the 2024–2025 Audit Programme.

Aim and objectives were to improve the management of patients undergoing seclusion at North Staffordshire Combined Healthcare NHS Trust (NSCHT), and to assess compliance with seclusion standards and identify actions for improvement where necessary.

**Methods:** All seclusions were identified by the Mental Health Law Team (n=46) from January 2023 to February 2024. Data were collected using a template based on standards and analysed with SPSS by the Clinical Audit Department.

**Results:** Seclusions mostly occurred within the first week of admission, mainly in patients with bipolar disorder or paranoid schizophrenia, with over half detained under Section 2 of the MHA. The majority were authorised by the Nurse in Charge, with incident forms completed in 83% and de-escalation attempted in 87%. NEWS (National Early Warning Score) was incomplete in 82% at seclusion initiation. The Site Manager was informed in all cases, but timings were unclear in 93.5%. The doctor was notified in all but one case (67% within 30 minutes).

100% had medical reviews, with 39% within one hour and 29% after 120 minutes. In 96% of cases, reviews were repeated before Multi-disciplinary Team (MDT) meetings, with half occurring every four hours. Nursing reviews occurred in all cases, with half conducted every two hours and two nurses involved in two-thirds of cases.

Internal and external MDTs took place in 63% and 72% of cases, respectively. Internal MDTs occurred in 17% of cases in the first 24 hours, and external MDTs in 46%. External MDTs were repeated every 24 hours in 83%. In 46% of cases, both internal and external MDTs were conducted, but 11% had neither. Night-time reviews were suspended in 14 cases, as 10 patients were asleep, leading to review deferral.

The reason for ongoing seclusion was stated in all but one case. Termination reasons were reported in 91% of cases, with 78% showing required steps undertaken.

**Conclusion:** This audit identifies strengths in authorisation, reporting, and de-escalation, with areas for improvement in review timing, NEWS assessments, and MDT consistency. Recommendations, shared with stakeholders, are in progress, including staff training, policy updates, automated reminders, enhanced documentation, Non-touch NEWS and virtual MDT meetings, to be monitored in the re-audit.

